# 

**Published:** 1921-05

**Authors:** 


### ANNOUNCEMENTS

*National Dental Association* will hold its general annual meeting in Milwaukee, the week of August 15 to 19. Other national and special meetings will be held during this week, and in the same city. Special announcement will be issued soon.

### SPECIAL NOTICE

The Bonwill Mechanical Mallet.—For the purpose of forwarding a revival of foil fillings at the coming meeting of the National Dental Association in Milwaukee in August, will any dentist who has had experience in the use of the Bonwill Mechanical Mallet (now issued as the S. S. White Engine Mallet No. 3), and who knows the Bonwill technique in condensing gold foil with that instrument, kindly send his name and address to Dr. Charles Southwell, 409 Goldsmith Building, Milwaukee, Wis.

